# CD47/SIRPα pathway mediates cancer immune escape and immunotherapy

**DOI:** 10.7150/ijbs.60782

**Published:** 2021-07-25

**Authors:** Xiao Jia, Bingjun Yan, Xiaoqing Tian, Qian Liu, Jianhua Jin, Juanjuan Shi, Yongzhong Hou

**Affiliations:** 1 Department of Oncology, the Affiliated Wujin Hospital, Jiangsu University, Changzhou, Jiangsu Province, 213017, the People's Republic of China.; 2 School of Life Sciences, Jiangsu University, Zhenjiang, Jiangsu Province, 212013, the People's Republic of China.; 3 Department of Oncology, the Wujin Clinical College of Xuzhou Medical University, Jiangsu Province, 212017, the People's Republic of China.

**Keywords:** CD47, SIRPα, immune escape, innate immune response, adaptive immune response, cancer immunotherapy

## Abstract

The adaptive immune checkpoints such as PD-1(programmed death-1)/PD-L1 (programmed death-ligand 1) play an important role in cancer immunotherapy, whereas increasing evidence suggests that cancer cell evades immune surveillance by innate immune checkpoints such as SIRPα (signal-regulatory protein α)/CD47 (cluster of differentiation 47). In multiple types of cancer cells and solid tumor tissues, highly expressed CD47 protein level has been observed, which is triggered by some transcription factors including NFκB, Myc, and HIF. As a transmembrane protein, the binding of CD47 to SIRPα ligand on phagocytes results in phagocytosis resistance and cancer cell immune escape. In contrast, CD47-SIRPα interaction blockade enhances cancer cell clearance by phagocytes such as macrophages and dendritic cells (DCs) to activate an innate immune response, whereas this process could promote antigen cross-presentation by antigen present cells (APCs) leading to T cell priming, consequently, activates an adaptive antitumor immune response. In this review, we discussed the current SIRPα-CD47 axis-mediated cancer cell immune escape and immunotherapy, which could provide an effective antitumor strategy by the innate and adaptive immune response.

## Introduction

**Figure 1:**
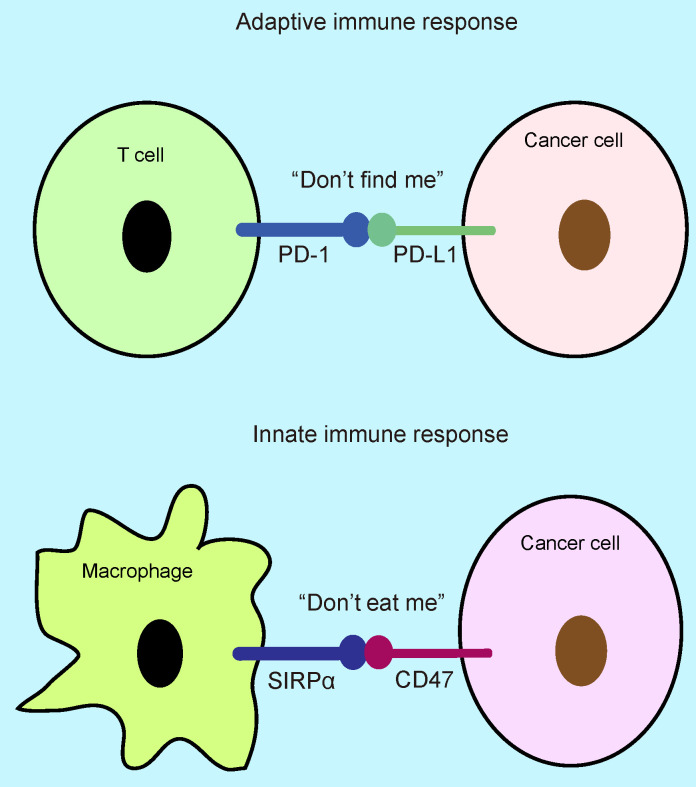
** “Don't eat me” and “don't find me” signal.** The binding of CD47 to SIRPα on phagocytes inhibits phagocytosis, which functions as “don't eat me” signal, whereas the binding of PD-L1 to PD-1 serves as “don't find me” signal that inhibits T cell killing. APCs: antigen present cells.

The innate and adaptive immune systems of host play an important role in killing cancer cells and inhibiting tumor progression 1-3, while cancer cell exhibits immune escape by expression of some immune checkpoint proteins such as PD-L1 (programmed death-ligand 1) and CD47 (cluster of differentiation 47) 3, 4. PD-1(programmed death-1)/PD-L1 checkpoint functions as “don't find me” signal to the adaptive immune response 5-7, whereas SIRPα (signal-regulatory protein α)-CD47 axis serves as “don't eat me” signal to the innate immune response 7, 8 (Figure 1). The interaction of PD-L1 with surface PD-1 receptor on T cells leads to inhibition of cancer cell killing 9, 10, whereas the binding of CD47 to surface SIRPα receptor on phagocytes inhibits cancer cell clearance 7, 8, 11. CD47 is a widely expressed glycoprotein in normal and cancer cells with five transmembrane domains 12, 13, which binds to the extracellular domain of SIRPα on phagocytes leading to inhibition of phagocytosis 7, 12. The SIRPα/CD47 checkpoint was first identified in 1999 14, 15, which suppresses phagocytosis of phagocytes and promotes cancer immune escape 8, 16, 17. The clinical analysis shows that CD47 is highly expressed on multiple types of cancer patients including glioblastoma, ovarian, breast, bladder, colon, and hepatocellular carcinoma, which correlates with low survival 18. CD47 expression is trigged by multiple transcriptional factors including NFκB, Myc, and HIF, etc 3, 11, 19, 20, while the SIRPα-CD47 axis blockade enhances phagocytosis by macrophages and DCs to activate innate immune response resulting in tumor regression 3, 7, 11, 12, 18, whereas the phagocytosis by DCs activates DNA-sensing cGAS-STING-INF-γ-mediated adaptive immune response leading to T cell priming 21-23, suggesting that inhibition of SIRPα-CD47 axis could enhance innate and adaptive antitumor immune response. In this review, we discussed the regulatory mechanism of CD47-mediated cancer immune escape and immunotherapy.

## SIRPα-CD47 axis protects cancer cell from phagocytosis

**Figure 2:**
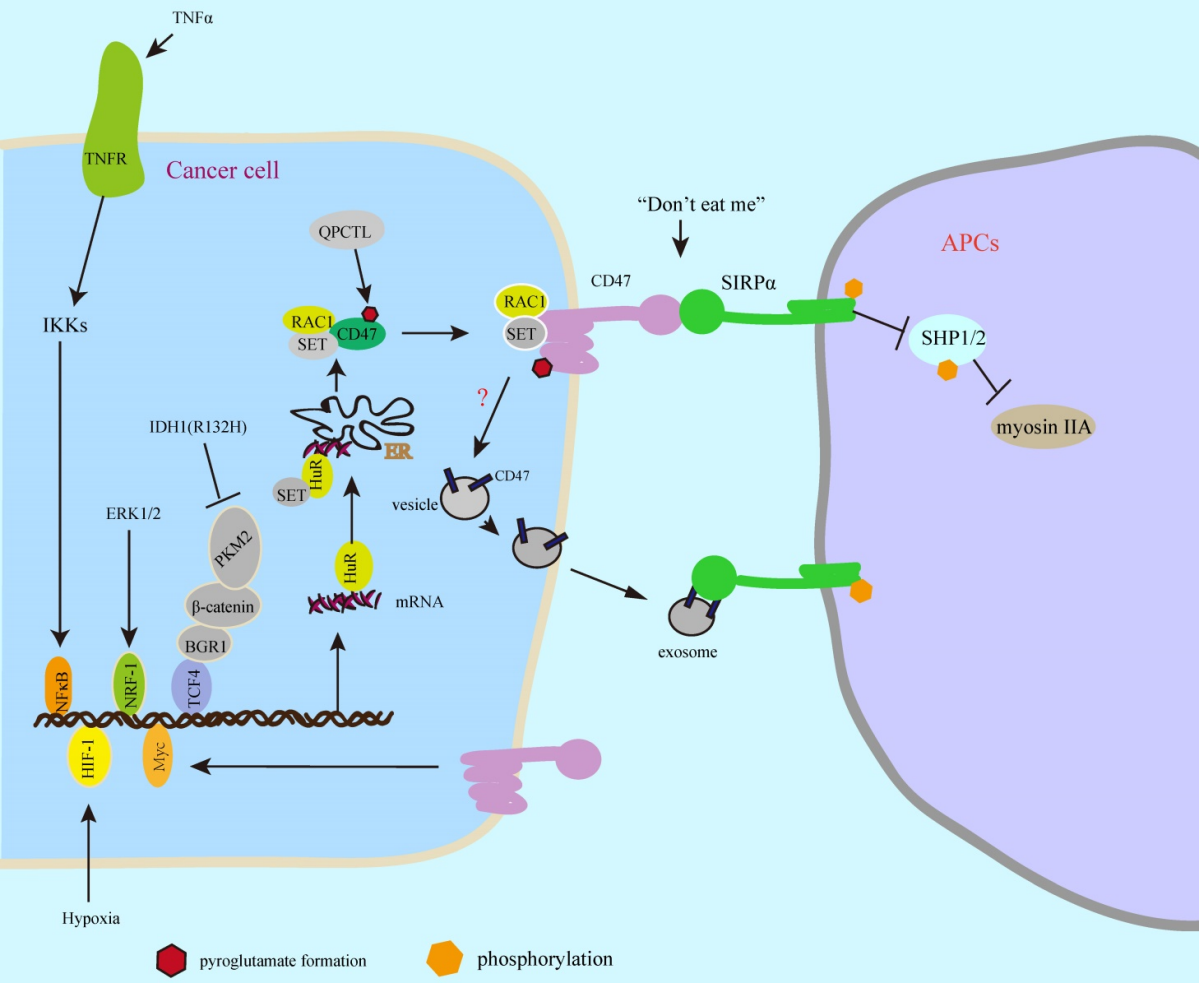
** SIRPα**-**CD47 axis protects cancer cell from phagocytosis.** Multiple transcription factors regulate CD47 expression in response to intracellular oncogenic activation pathways or extracellular stimuli. The new synthesized CD47 protein is delivered to the cellular surface by binding to SET/RAC complex proteins, which undergoes pyroglutamate formation by cyclotransferase-like (QPCTL) shortly after biosynthesis leading to increased phagocytosis resistance. In addition, the CD47 protein on the surface of the exosomes inhibits phagocytosis.

SIRPα is one of the SIRP family members, which was first identified in 1997 24. Thibaudeau et al 25 reports that SIRPα is highly expressed on macrophages. After that, CD47 was identified as the first ligand of SIRPα 14, 15. The binding of SIRPα to CD47 triggers SIRPα phosphorylation of ITIMs (immunoreceptor tyrosine-based inhibitory motifs) resulting in deactivation of myosin IIA, which is a critical step to block phagocytosis 16. CD47 glycoprotein is highly expressed in multiple types of cancer cells and human tumor tissues 7, 18, 26, 27, which is regulated by Myc oncogene 11. In this study, Myc directly binds to CD47 promoter and triggers its gene expression. In T cell acute lymphoblastic leukemia (T-ALL) xenograft tumor model, activation of Myc leads to tumor growth and inhibition of phagocytosis, which is alleviated by Myc inactivation 11. Interestingly, another report shows that silenced CD47 reduces Myc expression in oral squamous cell carcinoma 27, which suggests that CD47 could increase Myc expression. However, it is unclear whether CD47-Myc-CD47 feedback signal could regulate CD47 expression. In addition to direct regulation of CD47 promoter by Myc binding 11, extracellular stimuli also trigger CD47 gene expression 20, 28. In response to TNFα, activated NFκB (nuclear factor- κB) directly binds to a specific constituent enhancer of CD47 and increases its gene expression in MCF-7 breast cancer cells resulting in tumor growth by inhibiting phagocytosis 28. Moreover, under hypoxia condition, HIF-1 (hypoxia-inducible factor 1) binds to CD47 promoter and increases its expression resulting in inhibition of phagocytosis in breast cancer cells, which is a strong correlation between CD47 and HIF-1 by clinical analysis from thousands of breast cancer patients 20. The ChIP-Seq-based analysis shows that nuclear respiratory factor 1 (NRF-1) targets CD47 promoter 29. Consistent with this, oncogenic activation of ERK signal induces CD47 expression by NRF-1-mediated CD47 gene transcription in melanoma cells leading to inhibition of phagocytosis 30. In contrast, IDH1 (R132H) mutation in gliomas, negatively regulates CD47 gene transcription, which disrupts the binding of PKM2/β-catenin/BRG1 complex to TCF4 transcription factor resulting in inhibition of TCF4-mediated CD47 expression 31. In addition, Berkovits and Mayr 32 have described the mechanism of how does the new synthesized CD47 to be delivered to the cell surface. This study suggests that CD47 protein is present on the cell surface and intracellular, whereas the long 3'UTR of CD47 is critical for its surface localization. Mechanistically, the binding of HuR to long 3'UTR recruits SET to develop CD47 mRNA/HuR/SET complex and targets the endoplasmic reticulum (ER) surface, subsequently, the binding of SET to the new synthesized cytoplasmic domains of CD47 recruits RAC1 and forms a CD47/SET/RAC1 complex leading to the plasma membrane translocation of CD47 protein 32. Moreover, the expression of CD47 protein undergoes transcriptional modification by glutaminyl-peptide cyclotransferase-like (QPCTL), which induces CD47 pyroglutamate formation shortly after biosynthesis 17. In this study, it shows that the formation of pyroglutamate on CD47 enhances the binding of SIRPα to CD47, consequently, inhibits cancer cell clearance by phagocytes. In addition to present on cell surface, CD47 protein is observed on exosomes 33-35. Exosomes are extracellular vesicles (30-150 nm) with double-layer membrane, which is secreted from cells and effectively enter into other cells 36. High CD47 levels on the exosomes of breast cancer patients may be unfavourable 33, 34, and CD47 on the exosomes inhibits cancer cell clearance by phagocytes in pancreatic cancer 35, while it still unclear the secreted mechanism of CD47 on the exosomes. Taken together, CD47 gene expressions are regulated by multiple transcription factors, which could be transcriptional modification by pyroglutamate formation that enhances the binding of CD47 to SIRPα, consequently, inhibits phagocytosis by phagocytes and promotes cancer cell immune escape. CD47 on the exosomes also decreases antitumor activity by inhibition of phagocytosis. So, the surface CD47 on cancer cells and exosomes should be blocked for cancer immunotherapy (Figure 2).

## CD47 blockade enhances the innate and adaptive antitumor immune response

**Figure 3:**
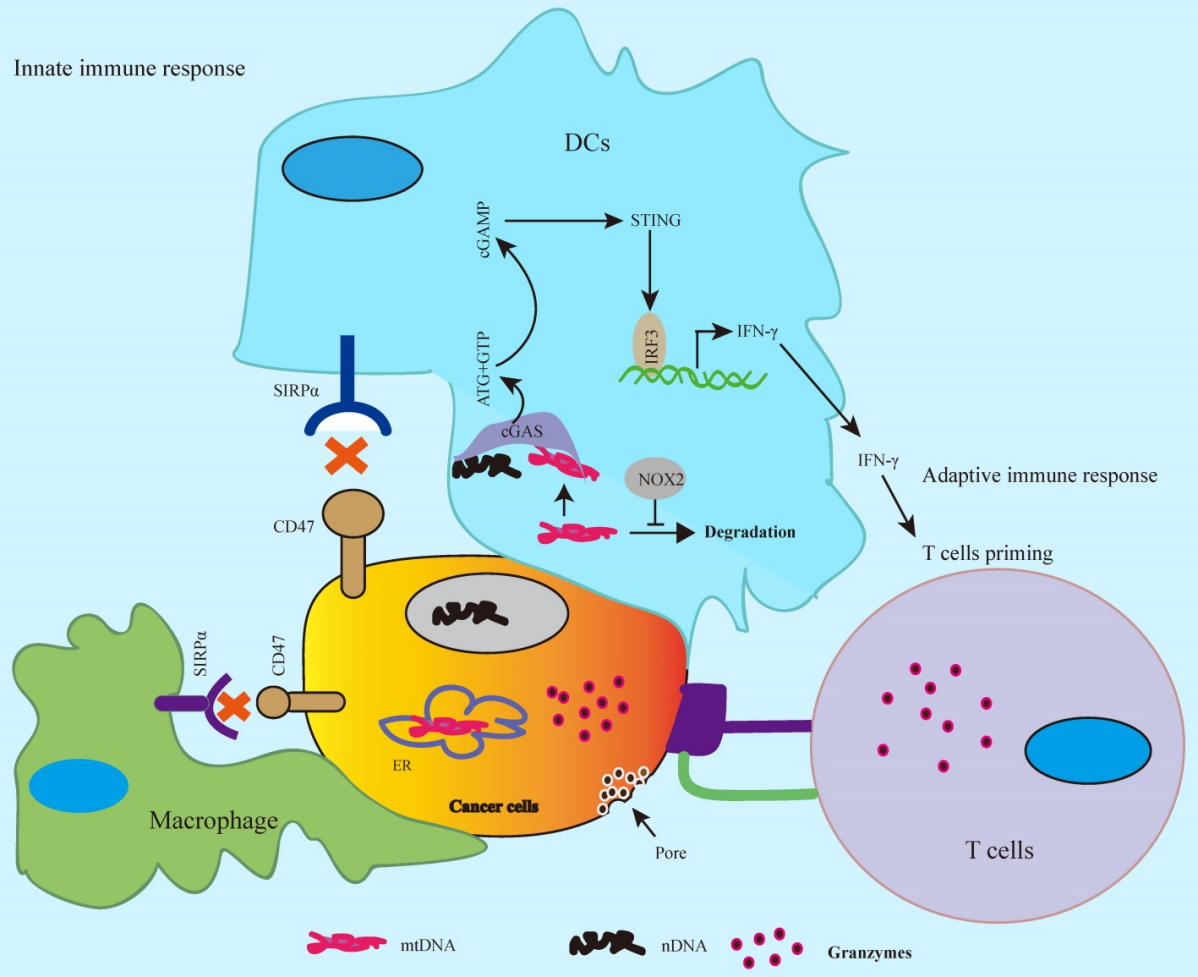
** CD47 blockade activates innate and adaptive antitumor immune response.** CD47-SIRPα axis blockade activates cGAS-cGAMP-STING-mediated innate immune response by tumor-derived nuclear DNA (nDNA) or mitochondrial DNA (mtDNA) in DCs, whereas the release of IFN-γ via cGAS-cGAMP-STING-INFR signal results in cytotoxic T cell priming and activates adaptive antitumor immune response.

CD47-SIRPα axis serves as “don't eat me” signal to the innate immune response 7, 8, whereas SIRPα-CD47 checkpoint blockade promotes phagocytosis by phagocytes such as macrophages and DCs leading to tumor regression by activation of innate immune response 23, 37, 38. The antitumor activity by CD47 blockade enhances cancer cell clearance by both of phagocytes and T cells 26, 37, and anti-CD47 antibody enhances CD8^+^ T cells killing but not CD4^+^ T cell in colon cancer cells 37. Silenced CD47 in T cells leads to enhanced T cell killing in irradiated melanoma cells 26. Vaccination with CD47 knockout tumor cells induces CD11c^+^SIRPα^+^ DCs activation and enhances T cell response in B16F0 melanoma mouse tumor model 39. As APCs, DCs engulf cancer cells and tumor-derived DNA in DCs activates cGAS (cGAMP), a cytosolic DNA sensor, subsequently, activates the downstream cGAS-cGAMP-STING innate immune response that exhibits antitumor activity 23, 40, whereas highly expressed CD47 inhibits this signaling pathway in cancer cells leading to tumor immune escape 7, 8. CD47-SIRPα blockade by anti-CD47 antibody enhances antigen cross-presentation by DCs and promotes T cell priming, consequently, CD8^+^ T cells, but not CD4^+^ T cells, mediate killing on colon cancer cells. In this process, cytosolic DNA sensor STING (Stimulator of interferon genes) is required for anti-CD47 antibody-mediated tumor regression 23. In addition, bispecific anti-PD-L1-SIRPα, both of SIRPα/CD47 and PD-1/PD-L1 checkpoints blockade, significantly enhances CD8^+^ T cell killing on colon cancer cells compared to SIRPα/CD47 or PD-1/PD-L1 blockade alone, which is involved in activation of STING-IFN-γ pathway in DCs 41. Combined CD47/SIRPα blockade with temozolomide in glioblastoma enhances phagocytosis and promotes T cell priming by activation of STING-IFN-γ pathway in DCs 38. In addition to tumor-derived DNA, the tumor mitochondrial DNA (mtDNA) can also trigger the cGAS-cGAMP-STING innate immune response 42. In this study, it reports that CD47 blockade leads to inhibition of degradation of tumor mtDNA by activation of NADPH oxidase NOX2 in DCs, consequently, activates the mtDNA-cGAS-STING-IFN-γ pathway in DCs. These findings suggest that CD47 blockade activates cGAS-cGAMP-STING-mediated innate immune response as well as adaptive immune response by cGAS-STING-IFN-γ signal-mediated T cell priming (Figure 3).

## SIRPα-CD47 checkpoint blockade in cancer immunotherapy

**Figure 4:**
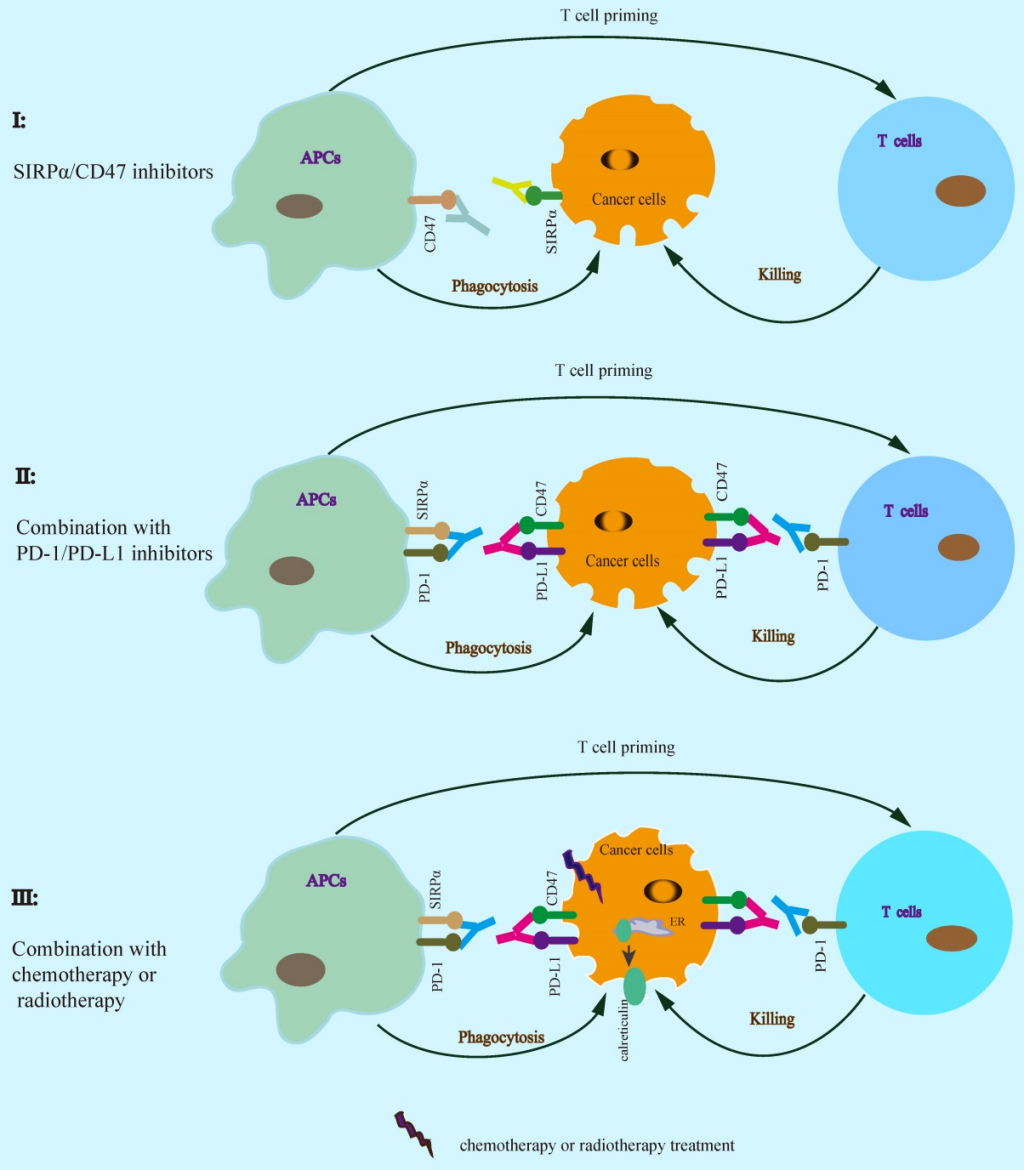
** SIRPα-CD47 checkpoint blockade enhances antitumor immunotherapy.** A rational combination of SIRPα-CD47 axis blockade contributes to enhancing the efficacy of cancer immunotherapy.

Increasing evidence suggests that SIRPα-CD47 checkpoint blockade enhances the efficacy of cancer immunotherapy (Table 1). SIRPα-CD47 axis blockade by using anti-CD47 antibody significantly enhances phagocytosis by macrophages and inhibits tumor growth 8, 18, 23, 26, 37, 43. Furthermore, SIRPα specific monoclonal antibody KWAR23 disrupts SIRPα-CD47 interaction resulting in inhibition of tumor growth by increasing phagocytosis 44. TTI-621 (SIRPαFc), a recombinant protein for CD47 binding, activates macrophage phagocytosis and inhibits tumor growth 45, 46. In addition to block CD47/SIRPα checkpoint alone, combined SIRPα/CD47 with PD-1/PD-L1 blockade enhances the efficiency of antitumor immunotherapy 41, 47, 48, suggesting that SIRPα-CD47 axis blockade could enhance PD-1/PD-L1 blockade therapy for cancer. The binding of the bispecific anti-PD-L1-SIRPα fusion protein to both of PD-L1 and CD47 on cancer cells significantly enhances antitumor activity in MC-38 colon cell xenograft tumor model 41. Similarly, anti-CD47 antibody synergizes with PD-L1 blockade for cancer immunotherapy in B16F10 melanoma tumor model 47. Moreover, combined with chemotherapy or radiotherapy also enhances the efficacy of cancer immunotherapy, which could increase T cell priming via the release of tumor-derived antigens consequent activation of APCs 21, 22. Similarly, cotrimoxazole synergizes with anti-CD47 antibody treatment leading to enhanced antitumor activity by both of phagocytosis and cGAS-STING DNA sensing signal 23. In response to mitoxantrone, anti-CD47 antibody significantly enhances antitumor activity in breast cancer cells 49. SIRPα-CD47 axis blockade enhances cancer cell clearance by phagocytes, which in turn promotes antigen cross-presentation by APCs resulting in enhanced T cell priming. Therefore, a rational combination of SIRPα-CD47 axis blockade contributes to cancer immunotherapy (Figure 4, Table 1).

## Conclusion

Highly expressed CD47 levels are present in multiple types of cancers including solid tumors and hematologic malignancies, which is major regulated by some transcription factors such as NFκB, Myc, HIF-1 and NRF-1. Although CD47 protein on exosomes has been observed, the mechanism of secreted pathway is still unclear. Given that normal and blood red cells are widely expressed CD47 that will limit the efficiency of anti-CD47 antibody therapy, therefore, a specific anti-CD47 antibody for CD47/SIRPα blockade is necessary. Especially, combined CD47/SIRPα with PD-1/PD-L1 checkpoints blockade will be preferred to inhibit cancer cell immune evasion. Since DNA damage stimuli could trigger an adaptive antitumor immune response by DNA-sensing cGAS-STING-INF-γ pathway or release of tumor-derived antigens, a rational combined anti-CD47 antibody with chemotherapy or radiotherapy could enhance the efficiency of antitumor immunotherapy.

## Figures and Tables

**Table 1:** SIRPα/CD47 blockade and antitumor immunotherapy

Targets	Tumor model	Reference
Anti-CD47+BRAF/MEK inhibitors	Melanoma	30
Anti-CD47	Acute myeloid leukemia (AML) stem cells	8
Anti-CD47	Breast cancer	49
KWAR23 (Anti-SIRPα)	Burkitt's lymphoma	44
TTI-621 (SIRPαFc)	Lymphoma. AML	46
Anti-PD-L1-SIRPα	Colon cancer	41
Anti-CD47+ anti-PD-L1	Melanoma	47
Cotrimoxazole+anti-CD47	Colon, B cell lymphoma	23
Mitoxantrone+anti-CD47	Breast cancer	49
1H9(anti-SIRPα)+anti-PD-L1	Melanoma	48
SRF23(anti-CD47)	Burkitt's lymphoma	43

## Abbreviations

PD-1: programmed death-1
PD-L1: programmed death-ligand 1
SIRPα: signal-regulatory protein α
CD47: cluster of differentiation 47
DCs: dendritic cells
APCs: antigen presenting cells
ITIMs: immunoreceptor tyrosine-based inhibitory motifs
T-ALL: T cell acute lymphoblastic leukemia
NFκB: nuclear factor- Κb
HIF-1: hypoxia-inducible factor 1
QPCTL: glutaminyl-peptide cyclotransferase-like
STING: Stimulator of interferon genes
